# Supplementary material for: Causal Relationship between Aging and Anorexia Nervosa: A White-Matter-Microstructure-Mediated Mendelian Randomization Analysis
Source: Biomedicines. 2024 Aug 16;12(8):1874. doi: 10.3390/biomedicines12081874 (PMC11351342; doi:10.3390/biomedicines12081874)
Supplement: Supplementary file 1 [file biomedicines-12-01874-s001.zip › Supplementary Figure/Figure S8/Leaveoneout plot of MR analysis for TL to ubm-b-1746.pdf]

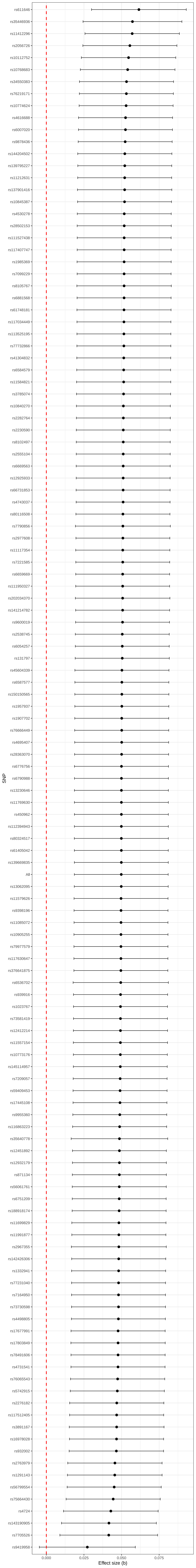
rs611646
rs35446936
rs11412296
rs2056726
rs10112752
rs10768683
rs34550383
rs76219171
rs10774624
rs4616688
rs6007020
rs9878436
rs144204502
rs139795227
rs11212631
rs137901416
rs10845387
rs4530278
rs28502153
rs111527438
rs117407747
rs1985369
rs7099229
rs8105767
rs6881568
rs61748181
rs117034449
rs113525195
rs77732866
rs41304832
rs6584579
rs11584821
rs3785074
rs10840270
rs2282764
rs2230590
rs8102497
rs2555104
rs6669563
rs12925933
rs66731853
rs4743037
rs80116508
rs7790856
rs2977608
rs11117354
rs7221585
rs6659669
rs111950327
rs202034370
rs141214782
rs9600019
rs2538745
rs6054257
rs131797
rs45604339
rs6587577
rs150150565
rs1957937
rs1907702
rs76666449
rs4695407
rs28363070
rs6776756
rs6790988
rs13230646
rs11769630
rs450962
rs112394943
rs80324517
rs61405042
rs139669835
A8
rs13062095
rs11579626
rs9398196
rs11085072
rs10905255
rs79977570
rs117630647
rs376641875
rs6536702
rs939916
rs1023767
rs73581419
rs12412214
rs11557154
rs10773176
rs145114957
rs7209057
rs59409453
rs17445108
rs9955360
rs116863223
rs35640778
rs12451892
rs12932179
rs871134
rs56061761
rs6751209
rs188918174
rs11699829
rs11991877
rs2967355
rs142426306
rs1332941
rs77231040
rs7164950
rs73730598
rs4498805
rs17677991
rs17803849
rs78491606
rs4731541
rs76065543
rs5742915
rs2276182
rs117512405
rs3891167
rs16978028
rs932002
rs2763979
rs1291143
rs56799554
rs75664430
rs4724
rs143190905
rs7705526
rs9419958
SNP
0.000
0.025
0.050
0.075
Effect size (b)
